# Comprehensive Analysis of *Ghd7* Variations Using Pan-Genomics and Prime Editing in Rice

**DOI:** 10.3390/genes16040462

**Published:** 2025-04-17

**Authors:** Jiarui Wang, Shihang Liu, Jisong Pu, Jun Li, Changcai He, Lanjing Zhang, Xu Zhou, Dongyu Xu, Luyao Zhou, Yuting Guo, Yuxiu Zhang, Yang Wang, Bin Yang, Pingrong Wang, Xiaojian Deng, Changhui Sun

**Affiliations:** 1 State Key Laboratory of Crop Gene Exploration and Utilization in Southwest China, Rice Research Institute, Sichuan Agricultural University, Chengdu 611130, China; wangjiarui1025@163.com (J.W.); lsh2372728743@163.com (S.L.); xcp106789331@163.com (J.P.); lee1808484677@163.com (J.L.); he15680693552@163.com (C.H.); zhanglanjing7@163.com (L.Z.); 17852981586@163.com (X.Z.); y18839161704@163.com (D.X.); 19162624732@163.com (L.Z.); yutingguo415@163.com (Y.G.); m15928770578@163.com (Y.Z.); yb18080897532@163.com (B.Y.); prwang@sicau.edu.cn (P.W.); 2 Panxi Crops Research and Utilization Key Laboratory of Sichuan Province, College of Agricultural Science, Xichang University, Liangshan 615000, China; wang_yang0707@163.com

**Keywords:** rice, pan-genome, heading date, *Ghd7*, structural variations, prime editor

## Abstract

The *Ghd7* gene in rice plays a crucial role in determining heading date, plant height, and grain yield. However, the variations in *Ghd7* and their functional implications across different rice accessions are not fully understood. Based on the release of a large amount of rice genome data in recent years, we investigated *Ghd7* through pan-genome analysis of 372 diverse rice varieties and figured out the structural variations (SVs) in the *Ghd7* locus. However, due to the high cost of pan-genomes, most genomes are based on next-generation sequencing (NGS) data now. Therefore, we developed a method for identifying SVs using NGS data and Polymerase Chain Reaction (PCR) based on the results of pan-genome analysis and identified 977 accessions carrying such SVs of *Ghd7*. Furthermore, we identified 46 single-nucleotide polymorphisms (SNPs) and one insertion-deletion (InDel) in the coding region of *Ghd7*. They are classified into 49 haplotypes. Notably, a splice-site mutation in haplotype H6 causes aberrant mRNA splicing. Using prime editing (PE) technology, we successfully restored the functional of *Ghd7* in Yixiang 1B (YX1B), delaying the heading date by approximately 16 days. This modification synchronized the heading date between YX1B and the restorer line Yahui 2115 (YH2115R), enhancing the hybrid rice seed production efficiency. In conclusion, our findings highlight the potential of integrating pan-genomics and precision gene editing to accelerate crop improvement and enhance agronomic traits.

## 1. Introduction

Over the past two decades, technological advancements in sequencing and assembly have driven the rapid growth and massive expansion of plant genomics [1,2,3,4]. The emergence of third-generation sequencing platforms, such as Pacific Biosciences (Menlo Park, CA, USA) (PacBio) and Oxford Nanopore Technologies (Oxford, UK) (ONT), has further revolutionized genome assembly by producing long reads that, when combined with the accuracy of next-generation sequencing (NGS), facilitate the construction of highly contiguous and accurate genomes [5,6,7,8,9,10]. However, the availability of multiple high-quality genomes has revealed a critical limitation: a single reference genome is insufficient to capture the full genetic diversity of a species due to the extensive structural and sequence variations among accessions [11,12].

To address this limitation, the concept of the “pan-genome” was introduced. Pan-genomes represent the genomic diversity of a species and include core genes, found in all individuals, as well as variable genes, which are absent in some individuals [11,13]. Since its inception, the pan-genome framework has been refined and applied to numerous crop species, including rice, soybean, maize, and wheat [14,15,16,17,18,19,20,21,22,23]. For example, the graph-based rice pan-genome, constructed from 33 genetically diverse accessions, not only uncovered extensive genomic variations but also provided insights into their roles in genome evolution, gene expression, and environmental adaptation [24]. Moreover, the construction of a super pan-genome based on 251 high-quality rice genomes has further enhanced our ability to mine functional genes and utilize germplasm resources [25]. In soybeans, a pan-genome map was constructed by resequencing the genomes of 2898 samples and selecting 26 representative samples for genome assembly. This greatly promoted the study of soybean evolution and functional genomics [26]. In wheat, 17 representative varieties were selected from over 3500 nationally approved varieties bred in China. High-quality genomes were assembled at the chromosome level, and nearly 250,000 structural variations (SVs) were accurately identified. It also presented the history of wheat breeding in China since the 1950s at the genomic level, revealed the influence of genomic structural variation on wheat adaptability and major variety formation, and provided a new perspective and strategy for future wheat design breeding [27]. In maize, the construction of 12 genomes and analysis of heterosis mechanisms using pan-genomic and expression quantitative trait Loci (eQTL) approaches provided an important theoretical basis and genetic resources for accelerating maize improvement and breeding [28]. In diploid potatoes, the genomes of 31 representative germplasm were assembled from scratch, yielding a total of 60 haplotype assemblages. A phased pan-genome was constructed to identify a large number of genetic variants and analyze the wide genetic diversity of diploid potato germplasm [29]. In millet and setaria, 110 high-quality genomes were assembled from scratch. The first variation map of the *Setaria* and the first high-quality map genome of multigrain crops were constructed. Several loci and key genes related to millet domestication and breeding improvement were identified, and the genetic basis of millet domestication and improvement, as well as the role of map genomes in genetics and breeding applications, were systematically analyzed [30].

Compared with the traditional single reference genome, the pan-genome can reveal the genetic diversity of species more comprehensively, particularly in the study of SV, where it can identify a broader spectrum of structural changes [31], such as the 1.8 kb insertion in the first exon of *RFT1* (*LOC_Os06g06300*) [25,32], the 1116 bp deletion in the *DTH8* gene (*LOC_Os08g07740*) [33], and the 17.1 kb copy number variation (CNV) of *GL7* (*LOC_Os07g41200*) [34]. However, the functional characterization of these SVs is often hampered by the lack of high-resolution phenotypic data and scalable validation platforms. For instance, despite large-scale studies like the rice super pan-genome [25] having mapped thousands of SVs, fewer than 5% of these variants have been experimentally linked to agronomic traits, leaving a vast reservoir of unexplored genetic potential. Moreover, the integration of pan-genomic insights into breeding programs faces systemic barriers, including the computational complexity of analyzing graph-based genomes and the limited accessibility of these tools in resource-poor regions [35]. Even when functional variants are identified, translating them into practical solutions, such as designing haplotype-specific breeding markers or editing targets, requires interdisciplinary collaboration that is still underdeveloped [36]. These limitations underscore an urgent need to bridge pan-genomics with advanced functional genomics tools, such as prime editing and high-throughput phenotyping, to unlock the full value of genetic diversity for trait optimization [37].

Addressing these challenges, we focused on *Ghd7*, a pivotal gene that exemplifies both the potential and limitations of pan-genomic studies in crop improvement. As a pleiotropic regulator of heading date, plant height, and grain yield, *Ghd7* serves as an ideal model to investigate how SVs and functional haplotypes shape agronomic traits across diverse environments [38]. Functional alleles such as *Ghd7*-1 and *Ghd7*-3 enable rice to exploit long growing seasons in tropical regions by delaying flowering under long-day conditions, whereas loss-of-function alleles such as *Ghd7*-0 and *Ghd7*-2 facilitate adaptation to temperate climates with shorter growth durations [38]. However, despite its agricultural significance, critical gaps persist in our understanding of *Ghd7* diversity. For instance, a 38.3 kb deletion spanning *Ghd7* in the elite hybrid parent Zhenshan 97 (ZS97) was identified over a decade ago, yet the structural complexity of this deletion and its functional consequences remain unresolved, primarily due to the historical lack of high-quality genomic resources [38]. This case underscores a broader issue in pan-genomics: while large-scale SV detection is now feasible, linking specific SVs to phenotypic outcomes and applying these findings in breeding requires targeted integration of genomic tools and experimental validation [37].

In this study, we leveraged pan-genomic data from 372 high-quality rice genomes to systematically investigate *Ghd7* SVs and other various mutation sites, along with their functional implications. By developing a novel computational pipeline that combines short-read sequencing depth analysis with a low-cost Polymerase Chain Reaction (PCR)-based validation method, we identified 997 accessions carrying large fragment deletions in *Ghd7* from a dataset of 10,548 rice germplasms [39]. Furthermore, haplotype analysis of 3637 accessions revealed 46 single-nucleotide polymorphisms (SNPs) and one insertion-deletion (InDel), many of which were previously unreported. Among these, the splicing-site mutation (A to C) in the maintainer line Yixiang 1B (YX1B) stood out as a critical variant that disrupts *Ghd7* function by causing aberrant mRNA splicing and premature termination of the CCT (CONSTANS, CO-like, and TOC1) domain. To validate its functional impact, we employed prime editor (PE) technology to precisely edit this mutation, restoring Ghd7 activity and delaying the heading date by approximately 16 days. This modification not only synchronized flowering between YX1B and the hybrid rice restorer line, enhancing seed production efficiency, but also established a proof-of-concept framework for functional validation of other *Ghd7* haplotypes. Our findings highlight the transformative potential of integrating pan-genomics with precision gene editing, offering a robust template for dissecting the genetic basis of agronomic traits and accelerating crop improvement through targeted interventions.

## 2. Materials and Methods

### 2.1. High-Quality Genomes and NGS Data Collection

We collected a comprehensive dataset of high-quality genomes and NGS data to investigate the SVs of the *Ghd7* gene in rice. The high-quality genomes were sourced from multiple studies [24,25,40,41,42,43,44,45,46,47,48], with a total of 372 high-quality genomes included after excluding those with low assembly quality (Table S1). Additionally, we obtained 10,548 NGS datasets from the rice super pan-genome information resource database (RiceSuperPIRdb) [18,24,25,44,49,50,51,52,53,54,55,56,57,58,59,60,61,62].

### 2.2. Ghd7 SVs Identification

We downloaded the *Ghd7* sequence, including the 50 kb upstream and downstream chromosome fragments, from the Nipponbare (Nip) reference genome (Michigan State University, MSU; http://rice.plantbiology.msu.edu/; accessed on 1 December 2022). For each of the 372 high-quality genomes, we used *Ghd7* sequences as probes for Basic Local Alignment Search Tool (BLAST) v2.12 searches against each genome assembly. We identified 44 assemblies lacking *Ghd7* sequence information. Eight high-quality genomes (ZS97, DG, D62, Y58S, II32, CN1, FS32, G46) from *Ghd7*-free materials were selected for detailed analysis. We used the BLAST-Like Alignment Tool (Blat) v35.1 [63] to identify homologous sequences flanking *Ghd7*, and the results were visualized using dot plots to represent the actual large fragment deletions.

### 2.3. Ghd7 Sequence Alignment and Variation Annotation

We performed multiple sequence alignment of all *Ghd7* homologous sequences using MEGA v12.0.11 [64] to identify variable sites. We annotated the variation sites using ANNOVAR v2019Oct24 [65] to identify non-synonymous codons, synonymous codons, splice-site variations, and premature terminations (Table S5).

### 2.4. Identification of Large Fragment Deletions

To determine the large fragment deletions, we aligned 8 NGS datasets (ZS97, DG, D62, Y58S, II32, CN1, FS32, G46), which were known to contain large fragment deletions, to the Nip reference genome and identified highly conserved regions flanking the *Ghd7* gene. Based on these conserved regions, we defined three key 400 bp intervals: a, b, and c. Regions a and c are located in the upstream and downstream conserved regions where large fragments are missing, respectively, while region b is at the junction of the second exon and intron of the *Ghd7* gene. Then, we calculated the average sequencing depths of these three intervals. By comparing the sequencing depth of interval b to those of intervals a and c, we identified the large fragment deletion in *Ghd7*.

### 2.5. Haplotype Analysis

Using the SNPs of the *Ghd7* gene region in the RiceSuperPIRdb, we removed all samples containing deletions or heterozygosity, leaving 3637 accessions. Haplotype typing was performed using the gengHapR v1.2.4 software package [66]. The phylogenetic trees of 49 haplotype SNPs were constructed using MEGA v11.0.13 [67].

### 2.6. Geographic Distribution

We collected and sorted the longitude and latitude data of rice materials [68]. Using R v4.3.2 language and the ggplot2 v3.5.2 package [69], we generated the world maps displaying dots of different colors to represent the geographic distribution of different *Ghd7* alleles.

### 2.7. Prime Editor Technology

The pegRNA is the addition of a reverse transcription template (RT) and primer binding site (PBS) sequence containing new genetic information to the 3′ terminal of single-stranded guide RNA (sgRNA). Nick target sequence: GTGCTCCCACAATATGACAT; Non-nick target sequence: CATTTGCTTATGCGTACATC; RT + PBS sequence (underscore PBS sequence) as AGTTTTGCAGATGTCATATTGTGGG. These fragments were constructed into the vector PE-P3 (containing M-MLV and Cas9n (H840A)) through primer amplification and carrier ligation experiments [70]. The complex promoter-sgRNA-RT-PBS-M-MLV-Cas9 (H840A) was formed. Rice callus was cultured from wild YX1B material. The constructed gene editing vector PE-P3 was transferred into Agrobacterium EHA105 by liquid nitrogen freeze–thaw conversion and then into rice callus by Agrobacterium tumefaciens [71]. Embryogenic callus containing Agrobacterium was screened and cultured on 50 mg/L hygromycin medium to obtain resistant callus, and then the T_0_ generation transgenic rice was regenerated.

### 2.8. DNA Extraction and PCR

The CTAB method was used for the isolation of genomic DNA from the fresh leaves of individual rice plants. The PCRs were conducted following the guidelines of the 2 × Taq PCR StarMix (Tsingke, Beijing, China). The PCR primers are shown in Table S3. The PCR products were examined by electrophoresis on a 0.8% (*w*/*v*) agarose gel at 120 V for 20 min. The target sequences were verified through Sanger sequencing.

### 2.9. Heading Date Phenotype Collection and Analysis

Heading date phenotype data were obtained from http://snp-seek.irri.org (accessed on 20 February 2023) [72] and https://ricerc.sicau.edu.cn/ (accessed on 20 February 2023) [24].

## 3. Results

### 3.1. Structural Variation in Ghd7 in High-Quality Genomes

**Figure 1:**
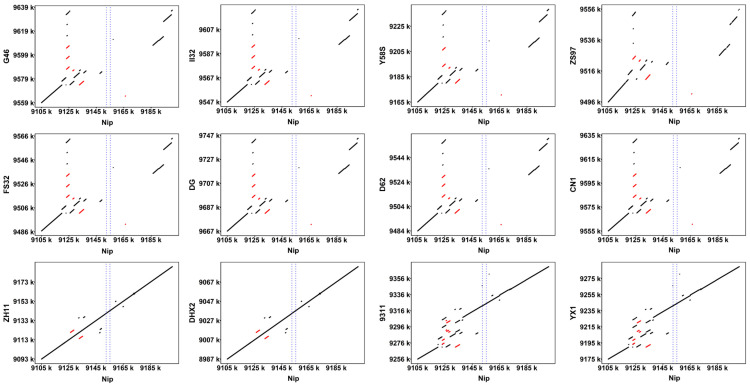
Dot plot of 12 materials compared to Nipponbare (Nip) sequences. The horizontal coordinate is the physical location of the Nip genome, and the vertical coordinate is the physical location of the genome of the comparison variety. The completeness of the long diagonal line in the middle is positively correlated with the matching degree of the two genomes. The scattered dots and short lines on both sides of the diagonal line represent homologous sequences with different physical locations, and the red scattered dots and short lines represent homologous sequences with inversion between the two. The blue dotted line represents the position of *Ghd7* in Nip. The more scattered points and short lines indicate that there are more homologous sequences with nonlinear relationships, and the greater the variation between varieties.

Identifying SVs based on short-read sequencing data is challenging and often unreliable [73]. To address this, we collected high-quality genomic data from 372 genetically diverse rice cultivars, including 338 *O*. *sativa* and 34 *O. rufipogon* samples. We detected pervasive presence/absence variation (PAV) containing *Ghd7* in 44 germplasms through sequence comparison analysis. To precisely define the deletion range of this PAV, we selected 8 high-quality genomes (ZS97, DG, D62, Y58S, II32, CN1, FS32, G46) with this variant for detailed analysis. By comparing homologous sequences flanking the *Ghd7* gene, we identified a about 60 kb deletion spanning the *Ghd7* region. Dot plot analysis revealed that this deletion was not continuous but intermittent, containing multiple types of SVs, including inversions, substitutions, and deletions, indicating complex chromosomal structural mutations at the *Ghd7* locus (Figure 1). Compared to Nip, *japonica* rice showed fewer SVs in this region, while *indica* rice exhibited more SVs, suggesting a key role in rice subspecies differentiation.

Furthermore, the *Ghd7* SVs are not completely identical, such as in ZS97 and CN1. Additionally, we observed a 1901 bp insertion SV in four materials (NH146, NH150, Aikoku, No8). Although both accessions exhibit a 60 kb deletion spanning the *Ghd7* region, the specific nature and complexity of the SVs differ. In ZS97, the deletion is part of a complex chromosomal structural mutation that includes multiple types of SVs, such as inversions, substitutions, and deletions. In contrast, CN1 may have a different pattern of SVs within the same region, even though it also carries a large fragment deletion. These differences highlight the diversity of *Ghd7* SVs across rice accessions and underscore the importance of detailed structural analysis in understanding the functional implications of these variations.

### 3.2. Identification of Large Fragment Deletions Using NGS Data

**Figure 2:**
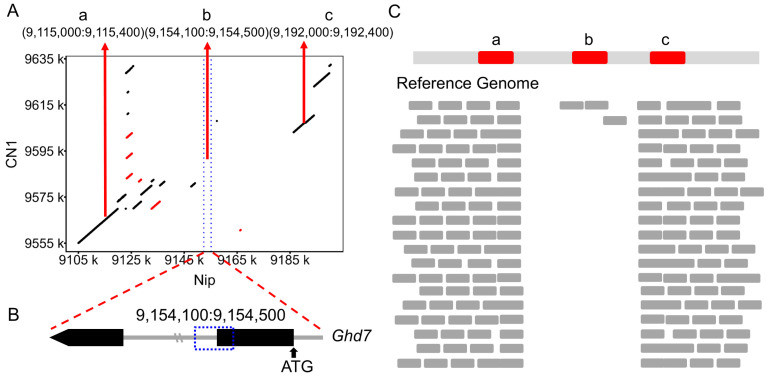
Identification of large block missing using short-read sequencing data. (**A**) The sequences of Nip and CN1 are compared in the dot plot, the horizontal coordinate is the sequence of Nip, the vertical coordinate is the sequence of CN1, the blue dotted line represents the position of *Ghd7* in Nip, and the three red arrows represent the three designed intervals of 400 bp length. The scattered dots and short lines on both sides of the diagonal line represent homologous sequences with different physical locations, and the red scattered dots and short lines represent homologous sequences with inversion between the two. (**B**) The two exons (indicated in the black rectangle) and the intron (indicated in the gray line) of *Ghd7* are shown in the graphics. The blue dotted rectangle indicates the position of interval b in *Ghd7*. (**C**) Comparison of short-read sequences mapping to different locations of the reference genome.

NGS data face significant challenges in the identification of SVs, particularly large fragment deletions, due to the short length of the sequencing reads. This limitation hinders the accuracy and reliability of SV detection. To address this issue, we developed a novel computational pipeline to identify large fragment deletions in the *Ghd7* gene using NGS data. We applied this method to 10,548 rice germplasms from the RiceSuperPIRdb [39] and successfully identified 977 accessions carrying the *Ghd7* deletion (Figure 2). We classified them as haplotype H0 (Table S2). This approach provides a new and effective strategy for SV identification using NGS data, overcoming the limitations of short-read lengths.

### 3.3. PCR-Based Validation of Large Fragment Deletions

To complement our computational approach for identifying large fragment deletions in the *Ghd7* gene, we designed a PCR-based method using two primer pairs: G1-F/R targeting the gene’s internal sequence and G2-F/R amplifying conserved flanking regions (Table S3). PCR amplification enables precise determination of large fragment deletions in the *Ghd7* region. When the *Ghd7*-containing fragment is deleted, G1 primers (targeting the coding sequence) fail to amplify a product, while G2 primers (anchored to conserved flanking regions) generate a distinct band. Conversely, if the *Ghd7* gene is present, G1 primers amplify the expected *Ghd7* fragment, while G2 primers show no amplification.

**Figure 3:**
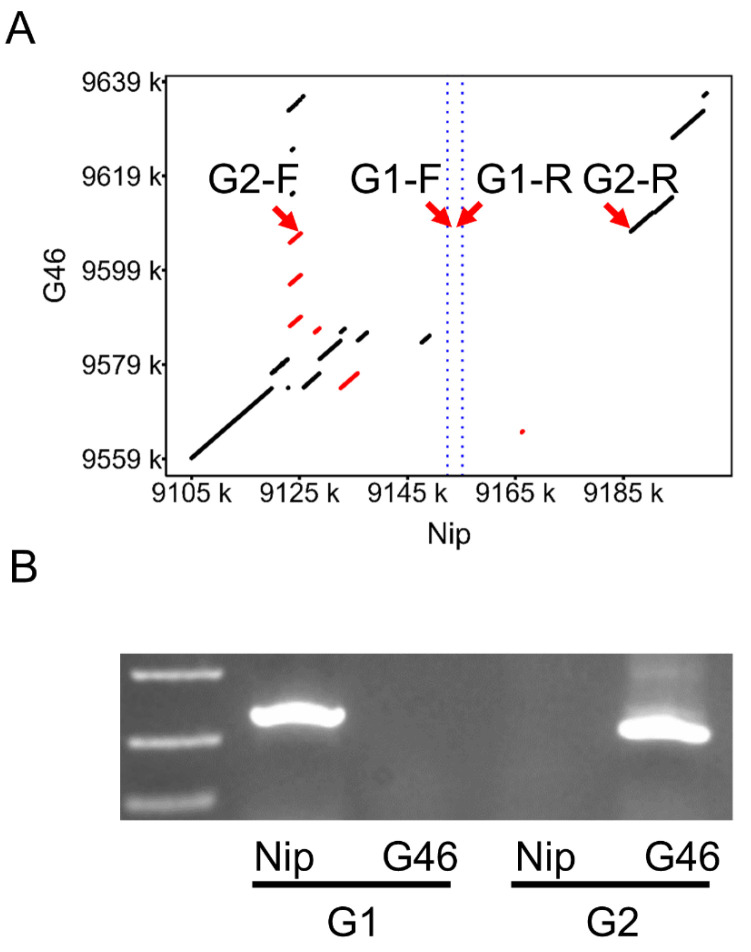
Identification of large fragment deletion by PCR. (**A**) Dot plot of G46 compared to Nip sequences. The red arrows indicate the locations of the two primers. The scattered dots and short lines on both sides of the diagonal line represent homologous sequences with different physical locations, and the red scattered dots and short lines represent homologous sequences with inversion between the two, the blue dotted line represents the position of *Ghd7* in Nip. (**B**) PCR results of the two primers of Nip and G46.

This PCR-based method provides a rapid, cost-effective strategy for validating large deletions in rice germplasms, demonstrating strong concordance with NGS results (Figure 3).

### 3.4. Haplotype Analysis of Ghd7

**Figure 4:**
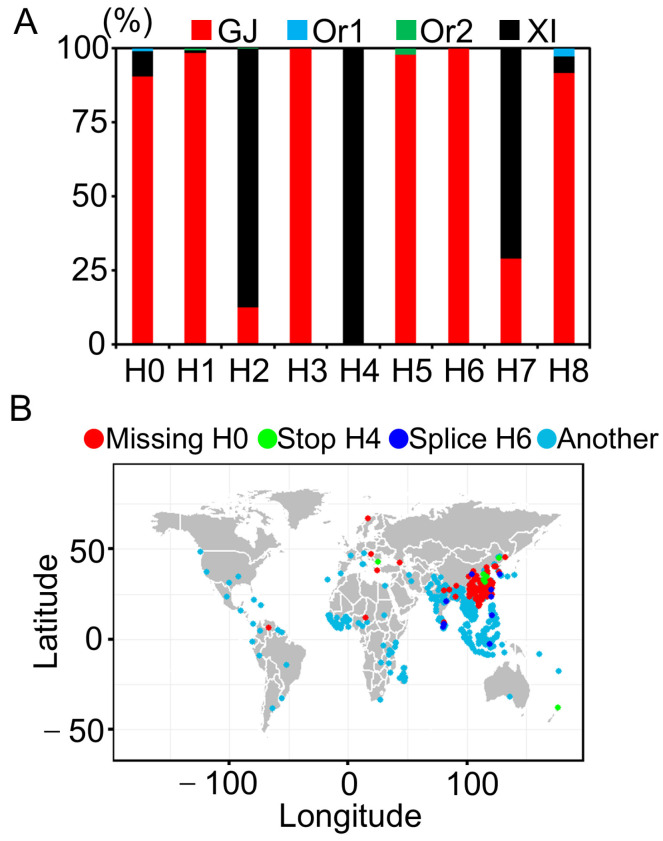
Haplotype distribution frequency and geographical distribution of *Ghd7*. (**A**) Distribution frequency of the *Ghd7* haplotype in different subspecies. GJ (*Japonica*), Or1 (*Oryza rufipogon1*), Or2 (*Oryza rufipogon2*), XI (*Indica*). (**B**) Geographical distribution of materials with different variant forms of *Ghd7*. The colored dots represent the classification of different variants.

The *Ghd7* gene coordinately regulates heading date and yield-related traits in response to photoperiod. Analyzing the natural allelic variations in the *Ghd7* gene in different rice germplasms can systematically reveal the mechanism of its coordinated regulation of photoperiod response, heading date, and yield-related traits, and provide key targets for molecular design breeding. Given the rapid development of rice germplasm resources and genomic sequencing, previous analyses of *Ghd7* variation types have become insufficiently comprehensive. Therefore, we conducted a comprehensive and systematic analysis of *Ghd7* genomic variations. Building on the identification of large fragment deletions, we performed a comprehensive haplotype analysis of the *Ghd7* gene using 10,548 NGS datasets from rice germplasm resources. After excluding samples with missing data or heterozygosity, we obtained 3637 high-quality germplasm datasets. We identified 211 SNPs in the *Ghd7* genomic sequence and excluded those located in synonymous codon positions or introns. Ultimately, we identified 46 SNPs and one InDel variant, including 42 nonsynonymous codon variants, one insertion at a conserved amino acid site, two splicing site variants, and two premature stop mutations (Table S4). Haplotype analysis revealed that *indica* rice subgroups were primarily distributed in haplotypes H0, H1, H3, H5, H6, and H8, while *japonica* rice subgroups were mainly in H2, H4, and H7. Splicing site variations were found in *indica* rice varieties, while premature stop mutations were detected in *japonica* rice varieties, indicating distinct selective pressures on *Ghd7* in different rice subspecies (Figure 4A).

### 3.5. Geographical Distribution of Ghd7 Haplotypes

The geographic distribution of *Ghd7* haplotypes reveals how natural allelic variation aligns with regional adaptation. Based on the 49 haplotypes identified, we constructed a global distribution map of *Ghd7* variants, including loss-of-function alleles (e.g., splice-site and premature termination mutations) and functional haplotypes (Figure 4B). The results showed that deletion, termination, and splicing site variants were distributed across different regions. Deletion types were present in *indica*, *japonica*, and *aus* subspecies, indicating widespread distribution. Splicing site variants were predominantly found in Southeast Asia, China, India, and Iraq, mainly in *indica* rice. Premature stop mutations were primarily in Northeast China and Jiangsu, all in temperate *japonica* varieties. The geographic distribution of *Ghd7* allelic diversity highlights its divergent roles in rice populations and demonstrates that natural variation in this gene is driven by ecological selection pressures. These findings provide a genetic foundation for tailoring rice varieties to specific climatic zones through allele-specific breeding strategies.

### 3.6. Validation of Splicing Site Variation and Improvement of Heading Date

**Figure 5:**
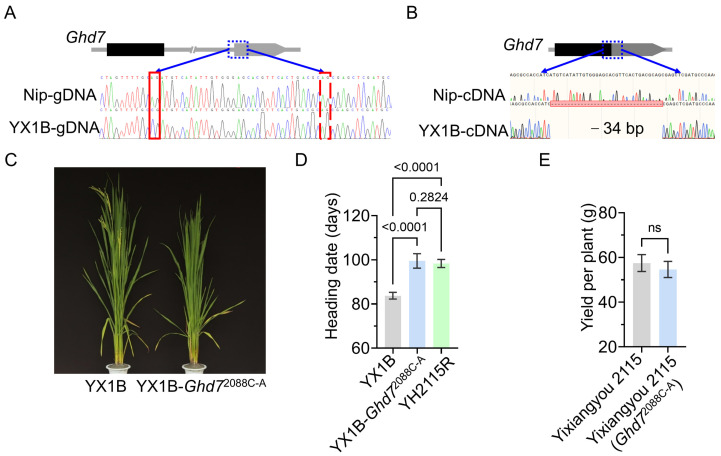
Detection and functional verification of splicing site variation. (**A**) *Ghd7* gene structure and *Ghd7* genome sequence comparison between Yixiang 1B (YX1B) and Nip. The blue dotted frame is the first 3’ splice site of the second exon of *Ghd7*. The two exons (the first exon is denoted by the black rectangle and the second by the gray rectangle) and the intron (indicated in the gray line) of *Ghd7* are shown in the graphics. The red frame is the splicing site identified by the Nip and the red dotted frame is the splicing site identified by YX1B. (**B**) *Ghd7* gene structure and *Ghd7* cDNA sequence comparison between YX1B and Nip. The two exons (the first exon is denoted by the black rectangle and the second by the gray rectangle) of *Ghd7* are shown in the graphics. (**C**) YX1B-*Ghd7*^2088C-A^ late heading phenotype. (**D**) Comparison of the heading date of YX1B, YX1B-*Ghd7*^2088C-A^, and YH2115R. (**E**) Comparison of yield per plant between hybrid rice Yixiangyou 2115 and Yixiangyou 2115 (*Ghd7*^2088C-A^). *p* values for One-Way ANOVA; (ns) no significance.

Building on the foundation of the numerous variant sites identified, we intend to utilize PE technology for validation. In this study, we targeted a splice site variant at genomic position 9,152,733 in haplotype H6. Compared to the reference genome Nip, YX1B exhibited a mutation where adenine (A) at the first 3′ splice site of the second exon of *Ghd7* was changed to cytosine (C). This mutation led to aberrant mRNA splicing, resulting in a 34 bp deletion and a frameshift mutation that caused premature termination of the CCT domain (Figure 5A,B). Although this site has been reported, its function has not been validated to date [74]. To validate the functional impact of this variant, we applied PE technology to edit the splice-site mutation in YX1B. Primers flanking the splice site were used to confirm successful prime editing in T_0_ transgenic lines. The edited plants exhibited a delayed heading date of approximately 16 days, confirming that the splice-site variant indeed causes early heading (Figure 5C).

One of the main constraints in hybrid rice seed production is the asynchronous heading date between the parental lines, which hinders the mechanization of seed production. This modification synchronized flowering between YX1B and the hybrid rice restorer line Yahui 2115 (YH2115R), enabling the same sowing and planting and enhancing seed production efficiency (Figure 5D). Yield comparisons between the edited hybrid rice Yixiangyou 2115 (*Ghd7*^2088C-A^) and the control Yixiangyou2115 showed no significant difference, indicating that the editing did not negatively affect yield (Figure 5E). This study demonstrates the potential of integrating pan-genomics and precision gene editing to improve agronomic traits and accelerate crop improvement. The successful editing of this splice-site variant in YX1B provides a new strategy for the improvement and application of heading date in rice varieties.

## 4. Discussion

The advancement of sequencing technology and the significant reduction in sequencing costs have revolutionized plant genomics, enabling the assembly of genomes for an increasing number of species [4]. The pan-genome, which encompasses all sequences for a species, including consensus sequences, large SVs, and small variations such as SNPs and InDels, has emerged as a critical tool for genomic research. A key challenge now is how to effectively utilize the pan-genome to mine genetic variants and present them graphically [12].

In this study, we leveraged high-quality genomes from 372 genetically diverse rice varieties to analyze natural allelic variation at the *Ghd7* locus, which plays a crucial role in rice ecological adaptation. Using Nip as the reference genome and through multiple sequence alignment (Table S5), we identified a total of 42 variation sites (excluding introns), including 13 insertion-deletions (InDels) and 27 nonsynonymous codons, as well as one premature termination mutation and one previously unreported splice-site mutation. Compared to analyses using NGS data alone, our pan-genomic approach identified more indels and confirmed most previously reported variants, demonstrating the reliability of the pan-genomic analysis. However, the limited number of high-quality genomes restricts the identification of specific variants, highlighting the need for more comprehensive pan-genomic resources to fully explore gene function.

Our SVs analysis revealed that 977 accessions harbor large fragment deletions in *Ghd7*, indicating that structural variants affecting this gene are widespread across diverse genetic backgrounds. This aligns with previous findings that allelic variation at *Ghd7* is a major contributor to natural variation in heading date among rice cultivars [38]. Notably, functional validation of the *Ghd7* H6 haplotype remains relatively scarce in crop species. Fortunately, PE accurately corrects single-base substitutions without introducing double-strand breaks, providing a powerful tool for confirming the phenotypic effects of subtle genomic alterations [70,75]. In this case, we succeeded in the restoration of the wild-type splicing site in H6 in the maintainer line YX1B, which led to a delay in the heading date by approximately 16 days, confirming the functional importance of the splice-site mutation in disrupting Ghd7 activity.

While our findings provide compelling evidence linking this haplotype H6 to the altered *Ghd7* heading date function, we acknowledge certain mechanistic limitations. Specifically, we did not assess Ghd7 protein abundance, subcellular localization, or downstream DNA-binding activity, all of which could provide additional support for the causal role of the identified variant. Previous studies have shown that proper nuclear localization and repression function of Ghd7 require an intact CCT domain [38]. It remains unclear whether the truncated protein resulting from aberrant splicing in YX1B is subject to proteolytic degradation, mislocalization, or loss of function. Future investigations employing Western blotting, confocal microscopy, or interactome profiling could elucidate the downstream consequences of such mutations at the protein level.

Another limitation is the underexplored role of regulatory variation in the promoter region of *Ghd7*. Transcription of *Ghd7* is tightly regulated by photoperiodic and circadian signals, and cis-regulatory elements within its promoter have been implicated in shaping diurnal expression patterns and adaptation to different latitudinal zones [76]. SVs or SNPs in these noncoding regulatory regions could modulate expression levels, timing, or epigenetic configuration, thereby influencing heading date independently of coding sequence variation. Integrating transcriptomic and epigenomic datasets, such as RNA-seq, ATAC-seq, or ChIP-seq, across different haplotypes would be a valuable next step in characterizing the transcriptional landscape and regulatory architecture of *Ghd7*.

Moreover, from a breeding perspective, our study demonstrates the practical utility of pan-genome-guided genome editing. Large fragment deletions and the splice site of H6 were primarily found in three-line and two-line sterile lines, while strong functional Ghd7 was concentrated in restorer lines. Varieties such as Kitaake, Chimao, and KY131 exhibit premature termination mutations, suggesting a breeding tendency for *Ghd7* haplotype selection. By correcting the *Ghd7* splice site of H6 in YX1B using PE, we synchronized its flowering with the restorer line YH2115R. This synchronization enables efficient mechanized seed production and enhances the efficiency of the hybrid seed set. This “genotype-by-editing” strategy, where pan-genomic variation informs precise genome editing interventions, could be broadly extended to other agronomic traits such as plant height, stress resistance, and yield components. Our work not only deepens the functional understanding of Ghd7 variation but also illustrates a translational approach to precision breeding through the convergence of pan-genomics and genome editing technologies.

## 5. Conclusions

In conclusion, our study demonstrates that leveraging high-quality genomes and extensive genomic data to analyze functional gene variants and validate them using PE technology is an effective approach for identifying superior allelic variations. This method not only provides a powerful tool for gene discovery and functional validation but also offers a template for future research. By integrating advanced genomic technologies with precise gene editing, our study highlights the potential to accelerate crop improvement and enhance agronomic traits. The successful application of this approach in the analysis and validation of *Ghd7* variants sets a precedent for similar studies in other crops and genes, paving the way for more efficient and targeted genetic enhancement in plant breeding programs.

## Data Availability

The original contributions presented in this study are included in the article/Supplementary Materials. Further inquiries can be directed to the corresponding authors.

## Supplementary Materials

The following supporting information can be downloaded at https://www.mdpi.com/article/10.3390/genes16040462/s1, Table S1: List of high-quality genomic information; Table S2: 4613 pieces of material information; Table S3: List of primers used in this study; Table S4: Haplotype analysis of *Ghd7*; Table S5: Haplotype analysis of high-quality genomes.

## References

[1] The Arabidopsis Genome Initiative (2000). Analysis of the genome sequence of the flowering plant *Arabidopsis thaliana*. Nature.

[2] Ouyang S., Zhu W., Hamilton J., Lin H., Campbell M., Childs K., Thibaud-Nissen F., Malek R.L., Lee Y., Zheng L. (2007). The TIGR Rice Genome Annotation Resource: Improvements and new features. Nucleic Acids Res..

[3] Marks R.A., Hotaling S., Frandsen P.B., VanBuren R. (2021). Representation and participation across 20 years of plant genome sequencing. Nat. Plants.

[4] Sun Y., Shang L., Zhu Q.H., Fan L., Guo L. (2022). Twenty years of plant genome sequencing: Achievements and challenges. Trends Plant Sci..

[5] Eid J., Fehr A., Gray J., Luong K., Lyle J., Otto G., Peluso P., Rank D., Baybayan P., Bettman B. (2009). Real-time DNA sequencing from single polymerase molecules. Science.

[6] Mikheyev A.S., Tin M.M.Y. (2014). A first look at the Oxford Nanopore MinION sequencer. Mol. Ecol. Resour..

[7] Koren S., Walenz B.P., Berlin K., Miller J.R., Bergman N.H., Phillippy A.M. (2017). Canu: Scalable and accurate long-read assembly via adaptive k-mer weighting and repeat separation. Genome Res..

[8] Wenger A.M., Peluso P., Rowell W.J., Chang P.C., Hall R.J., Concepcion G.T., Ebler J., Fungtammasan A., Kolesnikov A., Olson N.D. (2019). Accurate circular consensus long-read sequencing improves variant detection and assembly of a human genome. Nat. Biotechnol..

[9] Cheng H., Concepcion G.T., Feng X., Zhang H., Li H. (2021). Haplotype-resolved de novo assembly using phased assembly graphs with hifiasm. Nat. Methods.

[10] Niu S., Li J., Bo W., Yang W., Zuccolo A., Giacomello S., Chen X., Han F., Yang J., Song Y. (2022). The Chinese pine genome and methylome unveil key features of conifer evolution. Cell.

[11] Tettelin H., Masignani V., Cieslewicz M.J., Donati C., Medini D., Ward N.L., Angiuoli S.V., Crabtree J., Jones A.L., Durkin A.S. (2005). Genome analysis of multiple pathogenic isolates of Streptococcus agalactiae: Implications for the microbial “pan-genome”. Proc. Natl. Acad. Sci. USA.

[12] Bayer P.E., Golicz A.A., Scheben A., Batley J., Edwards D. (2020). Plant pan-genomes are the new reference. Nat. Plants.

[13] Golicz A.A., Bayer P.E., Bhalla P.L., Batley J., Edwards D. (2020). Pangenomics Comes of Age: From Bacteria to Plant and Animal Applications. Trends Genet..

[14] Hirsch C.N., Foerster J.M., Johnson J.M., Sekhon R.S., Muttoni G., Vaillancourt B., Peñagaricano F., Lindquist E., Pedraza M.A., Barry K. (2014). Insights into the Maize Pan-Genome and Pan-Transcriptome. Plant Cell.

[15] Li Y.-h., Zhou G., Ma J., Jiang W., Jin L.-g., Zhang Z., Guo Y., Zhang J., Sui Y., Zheng L. (2014). *De novo* assembly of soybean wild relatives for pan-genome analysis of diversity and agronomic traits. Nat. Biotechnol..

[16] Schatz M.C., Maron L.G., Stein J.C., Wences A.H., Gurtowski J., Biggers E., Lee H., Kramer M., Antoniou E., Ghiban E. (2014). Whole genome de novo assemblies of three divergent strains of rice, *Oryza sativa*, document novel gene space of *aus* and *indica*. Genome Biol..

[17] Montenegro J.D., Golicz A.A., Bayer P.E., Hurgobin B., Lee H., Chan C.-K.K., Visendi P., Lai K., Doležel J., Batley J. (2017). The pangenome of hexaploid bread wheat. Plant J..

[18] Wang W., Mauleon R., Hu Z., Chebotarov D., Tai S., Wu Z., Li M., Zheng T., Fuentes R.R., Zhang F. (2018). Genomic variation in 3,010 diverse accessions of Asian cultivated rice. Nature.

[19] Zhao Q., Feng Q., Lu H., Li Y., Wang A., Tian Q., Zhan Q., Lu Y., Zhang L., Huang T. (2018). Pan-genome analysis highlights the extent of genomic variation in cultivated and wild rice. Nat. Genet..

[20] Gao L., Gonda I., Sun H., Ma Q., Bao K., Tieman D.M., Burzynski-Chang E.A., Fish T.L., Stromberg K.A., Sacks G.L. (2019). The tomato pan-genome uncovers new genes and a rare allele regulating fruit flavor. Nat. Genet..

[21] Alonge M., Wang X., Benoit M., Soyk S., Pereira L., Zhang L., Suresh H., Ramakrishnan S., Maumus F., Ciren D. (2020). Major Impacts of Widespread Structural Variation on Gene Expression and Crop Improvement in Tomato. Cell.

[22] Walkowiak S., Gao L., Monat C., Haberer G., Kassa M.T., Brinton J., Ramirez-Gonzalez R.H., Kolodziej M.C., Delorean E., Thambugala D. (2020). Multiple wheat genomes reveal global variation in modern breeding. Nature.

[23] Hufford M.B., Seetharam A.S., Woodhouse M.R., Chougule K.M., Ou S., Liu J., Ricci W.A., Guo T., Olson A., Qiu Y. (2021). De novo assembly, annotation, and comparative analysis of 26 diverse maize genomes. Science.

[24] Qin P., Lu H., Du H., Wang H., Chen W., Chen Z., He Q., Ou S., Zhang H., Li X. (2021). Pan-genome analysis of 33 genetically diverse rice accessions reveals hidden genomic variations. Cell.

[25] Shang L., Li X., He H., Yuan Q., Song Y., Wei Z., Lin H., Hu M., Zhao F., Zhang C. (2022). A super pan-genomic landscape of rice. Cell Res..

[26] Liu Y., Du H., Li P., Shen Y., Peng H., Liu S., Zhou G.A., Zhang H., Liu Z., Shi M. (2020). Pan-Genome of Wild and Cultivated Soybeans. Cell.

[27] Jiao C., Xie X., Hao C., Chen L., Xie Y., Garg V., Zhao L., Wang Z., Zhang Y., Li T. (2025). Pan-genome bridges wheat structural variations with habitat and breeding. Nature.

[28] Wang B., Hou M., Shi J., Ku L., Song W., Li C., Ning Q., Li X., Li C., Zhao B. (2023). De novo genome assembly and analyses of 12 founder inbred lines provide insights into maize heterosis. Nat. Genet..

[29] Cheng L., Wang N., Bao Z., Zhou Q., Guarracino A., Yang Y., Wang P., Zhang Z., Tang D., Zhang P. (2025). Leveraging a phased pangenome for haplotype design of hybrid potato. Nature.

[30] He Q., Tang S., Zhi H., Chen J., Zhang J., Liang H., Alam O., Li H., Zhang H., Xing L. (2023). A graph-based genome and pan-genome variation of the model plant *Setaria*. Nat. Genet..

[31] Hu H., Zhao J., Thomas W.J.W., Batley J., Edwards D. (2025). The role of pangenomics in orphan crop improvement. Nat. Commun..

[32] Kojima S., Takahashi Y., Kobayashi Y., Monna L., Sasaki T., Araki T., Yano M. (2002). *Hd3a*, a rice ortholog of the *Arabidopsis FT* gene, promotes transition to flowering downstream of *Hd1* under short-day conditions. Plant Cell Physiol..

[33] Li D., Huang Z., Song S., Xin Y., Mao D., Lv Q., Zhou M., Tian D., Tang M., Wu Q. (2016). Integrated analysis of phenome, genome, and transcriptome of hybrid rice uncovered multiple heterosis-related loci for yield increase. Proc. Natl. Acad. Sci. USA.

[34] Wang Y., Xiong G., Hu J., Jiang L., Yu H., Xu J., Fang Y., Zeng L., Xu E., Xu J. (2015). Copy number variation at the *GL7* locus contributes to grain size diversity in rice. Nat. Genet..

[35] Della Coletta R., Qiu Y., Ou S., Hufford M.B., Hirsch C.N. (2021). How the pan-genome is changing crop genomics and improvement. Genome Biol..

[36] Sivabharathi R.C., Rajagopalan V.R., Suresh R., Sudha M., Karthikeyan G., Jayakanthan M., Raveendran M. (2024). Haplotype-based breeding: A new insight in crop improvement. Plant Sci..

[37] Li W., Liu J., Zhang H., Liu Z., Wang Y., Xing L., He Q., Du H. (2022). Plant pan-genomics: Recent advances, new challenges, and roads ahead. J. Genet. Genom..

[38] Xue W., Xing Y., Weng X., Zhao Y., Tang W., Wang L., Zhou H., Yu S., Xu C., Li X. (2008). Natural variation in *Ghd7* is an important regulator of heading date and yield potential in rice. Nat. Genet..

[39] Wang T., He W., Li X., Zhang C., He H., Yuan Q., Zhang B., Zhang H., Leng Y., Wei H. (2023). A rice variation map derived from 10 548 rice accessions reveals the importance of rare variants. Nucleic Acids Res..

[40] Mahesh H.B., Shirke M.D., Singh S., Rajamani A., Hittalmani S., Wang G.L., Gowda M. (2016). Indica rice genome assembly, annotation and mining of blast disease resistance genes. BMC Genom..

[41] Choi J.Y., Lye Z.N., Groen S.C., Dai X., Rughani P., Zaaijer S., Harrington E.D., Juul S., Purugganan M.D. (2020). Nanopore sequencing-based genome assembly and evolutionary genomics of circum-basmati rice. Genome Biol..

[42] Panibe J.P., Wang L., Li J., Li M.Y., Lee Y.C., Wang C.S., Ku M.S.B., Lu M.J., Li W.H. (2021). Chromosomal-level genome assembly of the semi-dwarf rice Taichung Native 1, an initiator of Green Revolution. Genomics.

[43] Song J.M., Xie W.Z., Wang S., Guo Y.X., Koo D.H., Kudrna D., Gong C., Huang Y., Feng J.W., Zhang W. (2021). Two gap-free reference genomes and a global view of the centromere architecture in rice. Mol. Plant.

[44] Zhang F., Xue H., Dong X., Li M., Zheng X., Li Z., Xu J., Wang W., Wei C. (2022). Long-read sequencing of 111 rice genomes reveals significantly larger pan-genomes. Genome Res..

[45] Zhang H., Wang Y., Deng C., Zhao S., Zhang P., Feng J., Huang W., Kang S., Qian Q., Xiong G. (2022). High-quality genome assembly of Huazhan and Tianfeng, the parents of an elite rice hybrid Tian-you-hua-zhan. Sci. China Life Sci..

[46] Zhang Y., Fu J., Wang K., Han X., Yan T., Su Y., Li Y., Lin Z., Qin P., Fu C. (2022). The telomere-to-telomere gap-free genome of four rice parents reveals SV and PAV patterns in hybrid rice breeding. Plant Biotechnol. J..

[47] Shang L., He W., Wang T., Yang Y., Xu Q., Zhao X., Yang L., Zhang H., Li X., Lv Y. (2023). A complete assembly of the rice Nipponbare reference genome. Mol. Plant.

[48] Wang Y., Li F., Zhang F., Wu L., Xu N., Sun Q., Chen H., Yu Z., Lu J., Jiang K. (2023). Time-ordering japonica/geng genomes analysis indicates the importance of large structural variants in rice breeding. Plant Biotechnol. J..

[49] Qiu J., Zhou Y., Mao L., Ye C., Wang W., Zhang J., Yu Y., Fu F., Wang Y., Qian F. (2017). Genomic variation associated with local adaptation of weedy rice during de-domestication. Nat. Commun..

[50] Gutaker R.M., Groen S.C., Bellis E.S., Choi J.Y., Pires I.S., Bocinsky R.K., Slayton E.R., Wilkins O., Castillo C.C., Negrão S. (2020). Genomic history and ecology of the geographic spread of rice. Nat. Plants.

[51] Lv Q., Li W., Sun Z., Ouyang N., Jing X., He Q., Wu J., Zheng J., Zheng J., Tang S. (2020). Resequencing of 1,143 indica rice accessions reveals important genetic variations and different heterosis patterns. Nat. Commun..

[52] Mao D., Xin Y., Tan Y., Hu X., Bai J., Liu Z.-y., Yu Y., Li L., Peng C., Fan T. (2019). Natural variation in the *HAN1* gene confers chilling tolerance in rice and allowed adaptation to a temperate climate. Proc. Natl. Acad. Sci. USA.

[53] Xia H., Luo Z., Xiong J., Ma X., Lou Q., Wei H., Qiu J., Yang H., Liu G., Fan L. (2019). Bi-directional Selection in Upland Rice Leads to Its Adaptive Differentiation from Lowland Rice in Drought Resistance and Productivity. Mol. Plant.

[54] Li X., Chen Z., Zhang G., Lu H., Qin P., Qi M., Yu Y., Jiao B., Zhao X., Gao Q. (2020). Analysis of genetic architecture and favorable allele usage of agronomic traits in a large collection of Chinese rice accessions. Sci. China Life Sci..

[55] Xiao N., Pan C., Li Y., Wu Y., Cai Y., Lu Y., Wang R., Yu L., Shi W., Kang H. (2021). Genomic insight into balancing high yield, good quality, and blast resistance of japonica rice. Genome Biol..

[56] Yano K., Yamamoto E., Aya K., Takeuchi H., Lo P.C., Hu L., Yamasaki M., Yoshida S., Kitano H., Hirano K. (2016). Genome-wide association study using whole-genome sequencing rapidly identifies new genes influencing agronomic traits in rice. Nat. Genet..

[57] Wang X., Wang W., Tai S., Li M., Gao Q., Hu Z., Hu W., Wu Z., Zhu X., Xie J. (2022). Selective and comparative genome architecture of Asian cultivated rice (*Oryza sativa* L.) attributed to domestication and modern breeding. J. Adv. Res..

[58] Higgins J., Santos B., Khanh T.D., Trung K.H., Duong T.D., Doai N.T.P., Hall A., Dyer S., Ham L.H., Caccamo M. (2022). Genomic regions and candidate genes selected during the breeding of rice in Vietnam. Evol. Appl..

[59] Chen W., Gao Y., Xie W., Gong L., Lu K., Wang W., Li Y., Liu X., Zhang H., Dong H. (2014). Genome-wide association analyses provide genetic and biochemical insights into natural variation in rice metabolism. Nat. Genet..

[60] Huang X., Wei X., Sang T., Zhao Q., Feng Q., Zhao Y., Li C., Zhu C., Lu T., Zhang Z. (2010). Genome-wide association studies of 14 agronomic traits in rice landraces. Nat. Genet..

[61] Huang X., Kurata N., Wei X., Wang Z.X., Wang A., Zhao Q., Zhao Y., Liu K., Lu H., Li W. (2012). A map of rice genome variation reveals the origin of cultivated rice. Nature.

[62] Zheng X., Pang H., Wang J., Yao X., Song Y., Li F., Lou D., Ge J., Zhao Z., Qiao W. (2022). Genomic signatures of domestication and adaptation during geographical expansions of rice cultivation. Plant Biotechnol. J..

[63] Kent W.J. (2002). BLAT--the BLAST-like alignment tool. Genome Res..

[64] Kumar S., Stecher G., Suleski M., Sanderford M., Sharma S., Tamura K. (2024). MEGA12: Molecular Evolutionary Genetic Analysis version 12 for adaptive and green computing. Mol. Biol. Evol..

[65] Wang K., Li M., Hakonarson H. (2010). ANNOVAR: Functional annotation of genetic variants from high-throughput sequencing data. Nucleic Acids Res..

[66] Zhang R., Jia G., Diao X. (2023). geneHapR: An R package for gene haplotypic statistics and visualization. BMC Bioinf..

[67] Tamura K., Stecher G., Kumar S. (2021). MEGA11: Molecular Evolutionary Genetics Analysis Version 11. Mol. Biol. Evol..

[68] Zhao H., Yao W., Ouyang Y., Yang W., Wang G., Lian X., Xing Y., Chen L., Xie W. (2015). RiceVarMap: A comprehensive database of rice genomic variations. Nucleic Acids Res..

[69] Ginestet C. (2011). ggplot2: Elegant Graphics for Data Analysis. J. R. Stat. Soc. Ser. A Stat. Soc..

[70] Xu W., Yang Y., Yang B., Krueger C.J., Xiao Q., Zhao S., Zhang L., Kang G., Wang F., Yi H. (2022). A design optimized prime editor with expanded scope and capability in plants. Nat. Plants.

[71] Hiei Y., Komari T. (2008). Agrobacterium-mediated transformation of rice using immature embryos or calli induced from mature seed. Nat. Protoc..

[72] Mansueto L., Fuentes R.R., Borja F.N., Detras J., Abriol-Santos J.M., Chebotarov D., Sanciangco M., Palis K., Copetti D., Poliakov A. (2017). Rice SNP-seek database update: New SNPs, indels, and queries. Nucleic Acids Res..

[73] Sedlazeck F.J., Rescheneder P., Smolka M., Fang H., Nattestad M., von Haeseler A., Schatz M.C. (2018). Accurate detection of complex structural variations using single-molecule sequencing. Nat. Methods.

[74] Sun K., Zong W., Xiao D., Wu Z., Guo X., Li F., Song Y., Li S., Wei G., Hao Y. (2023). Effects of the core heading date genes *Hd1*, *Ghd7*, *DTH8*, and *PRR37* on yield-related traits in rice. Theor. Appl. Genet..

[75] Lin Q., Zong Y., Xue C., Wang S., Jin S., Zhu Z., Wang Y., Anzalone A.V., Raguram A., Doman J.L. (2020). Prime genome editing in rice and wheat. Nat. Biotechnol..

[76] Zhou S., Zhu S., Cui S., Hou H., Wu H., Hao B., Cai L., Xu Z., Liu L., Jiang L. (2021). Transcriptional and post-transcriptional regulation of heading date in rice. New Phytol..

